# Widow spiders in the New World: a review on *Latrodectus* Walckenaer, 1805 (Theridiidae) and latrodectism in the Americas

**DOI:** 10.1590/1678-9199-JVATITD-2021-0011

**Published:** 2021-10-22

**Authors:** Marjolly Brigido Caruso, Pedro Santana Sales Lauria, Claudio Maurício Vieira de Souza, Luciana Lyra Casais-e-Silva, Russolina Benedeta Zingali

**Affiliations:** 1 Laboratory of Hemostasis and Venoms, Leopoldo de Meis Institute of Medical Biochemistry, Federal University of Rio de Janeiro (UFRJ), Rio de Janeiro, RJ, Brazil.; 2 Laboratory of Pharmacology and Experimental Therapeutics, School of Pharmacy, Federal University of Bahia (UFBA), Salvador, BA, Brazil.; 3 Laboratory of Arthropods, Vital Brazil Institute, Niterói, RJ, Brazil.; 4 Laboratory of Neuroimmunoendocrinology and Toxinology, Institute of Health Sciences, Federal University of Bahia (UFBA), Salvador, BA, Brazil.

**Keywords:** Latrodectus, Latrodectism, Widow spiders, Spider venom, Americas

## Abstract

Humankind has always been fascinated by venomous animals, as their toxic substances have transformed them into symbols of power and mystery. Over the centuries, researchers have been trying to understand animal venoms, unveiling intricate mixtures of molecules and their biological effects. Among venomous animals, *Latrodectus* Walckenaer, 1805 (widow spiders) have become feared in many cultures worldwide due to their extremely neurotoxic venom. The *Latrodectus* genus encompasses 32 species broadly spread around the globe, 14 of which occur in the Americas. Despite the high number of species found in the New World, the knowledge on these spiders is still scarce. This review covers the general knowledge on *Latrodectus* spp. from the Americas. We address widow spiders’ taxonomy; geographical distribution and epidemiology; symptoms and treatments of envenomation (latrodectism); venom collection, experimental studies, proteome and transcriptome; and biotechnological studies on these *Latrodectus* spp. Moreover, we discuss the main challenges and limitations faced by researchers when trying to comprehend this neglected group of medically important spiders. We expect this review to help overcome the lack of information regarding widow spiders in the New World.

## Background

Among the members of the Araneae order, *Latrodectus* spp. (widow spiders) are well known for constituting a hazard to humans. The envenomation by these spiders is marked by a massive neurotransmitter release that leads to neurotoxic manifestations of high morbidity. Although *Latrodectus* spp. represent only 1.3% of the biodiversity of the Theridiidae family, their extremely neurotoxic venom has transformed this small group of spiders into a major symbol of araneism in different cultures worldwide. The negative emotions evoked by these animals are not limited to folk beliefs and superstitions, but also permeate the scientific field, as shown by the terms that identify the genus itself (*Latrodectus* = thief, bandit, aggressor, hidden, sneaky) and one of its most studied species (*mactans* = killer, deadly).

Widow spiders have been the subject of several studies since the 18^th^ century, although there is still a lot to understand about these intricate animals due to the limitations found by researchers (e.g., little amounts of venom extracted, difficulty to keep the spiders in captivity, taxonomic instability, etc.). Around 44% of the validly recognized *Latrodectus* species can be found in the Americas, where they have been accountable for several cases of human envenomation [1, 2]. Nevertheless, little is known about the widow spiders from the American continent, as most of the early studies focused on the spiders from the Old World and there is currently no compilation of the knowledge about the American species. Therefore, this review covers the general knowledge on widow spiders, focusing on the species found in the Americas. Herein, we address widow spiders’ taxonomy; geographical distribution and epidemiology; symptoms and treatments of envenomation; venom collection, experimental studies, proteome and transcriptome; and biotechnological studies on *Latrodectus* spp.

## Taxonomy

Theridiidae (Sundevall, 1833) is one of the ten most diverse and widely distributed spider families on the planet, comprising 124 genera and 2510 species. Members of this family, known as the cobweb spiders, are greatly diverse regarding their morphology, ecology, and behavior. These spiders are distributed in seven subfamilies: Hadrotarsinae, Spintharinae, Pholcommatinae, Argyrodinae, Anelosiminae, Theridiinae, and Latrodectinae. The latter group is composed of four genera: *Asagena* Sundevall, 1833; *Crustulina* Menge, 1868; *Steatoda* Sundevall, 1833; and *Latrodectus* Walckenaer, 1805, which is considered by many authors as some of the most dangerous spiders for humans [3-6]. The first species of this genus was described in Italy by Rossi in 1790, as *Aranea 13-guttata.* It was later included in the *Latrodectus* clade by Walckenaer, who made the first correlation between the bites of these spiders and the neurotoxic syndrome known as latrodectism [3].

Despite the small number of *Latrodectus* species when compared to other spider groups, the taxonomy of widow spiders is considered complex and difficult [7]. This difficulty has been attributed to a wide range of factors: the low interest of experienced arachnologists to study this group deeply; the wide geographical distribution of many species and the great variations observed among their populations; and the traditional use of morphological characteristics in the description and identification of species. This scenario leads to instability in the group’s taxonomy, a critical issue for the correct identification of widow spiders. Consequently, it is harder to conduct studies on bio-ecology and toxinology, as well as to propose strategies of environmental surveillance for public health.

Numerous initiatives have been carried out for a taxonomic organization of this important group of spiders, some with regional coverage and others almost global in scope. It is possible to observe cycles of expansion and reduction in the number of valid *Latrodectus* species over time; each cycle is directly influenced by the methods available at the periods when the studies were conducted [8-11].

Initially, Bettini and Marolli [3] identified three different moments of the *Latrodectus* taxonomic trajectory. In the first phase, the number of species was increased as a result of adopting body color as the main systematic character [8]. The adoption of genitalia morphology as a central taxonomic parameter started a second stage, characterized by the strong decrease in valid species [10]. In the third phase, the number of species increased once again, based on the association of morphological knowledge and other biological and biochemical aspects of these spiders [12, 13]. The application of molecular biology tools for phylogenetic analysis of this genus in the mid-2000s [11] is a landmark that started the fourth phase in the knowledge of the biodiversity of *Latrodectus* spiders, consolidating the use of multidisciplinary bio-ecological approaches in the study of widow spiders.

The tree of phylogenetic relationships proposed by Garb *et al*. [11] distributes the species of *Latrodectus* into two broad monophyletic clades: (1) the *geometricus* clade, comprising *L. geometricus* and *L. rhodesiensis*, both species originated in the African continent; and (2) the *mactans* clade, comprising *L. mactans* and all other species from Africa, Israel, Spain, Australia, New Zealand, and the Americas. Surprisingly, these findings revive and corroborate the hypothesis of Levi [10], who had classified most species as synonyms of *L. mactans*, thereby attributing a semi-cosmopolitan character to this species. Also, the little genetic variation observed among *L. geometricus* populations from very distant locations indicates that the dispersion and introduction of this species in new territories may have occurred very recently, suggesting the influence of human activity.

The taxonomy of American *Latrodectus* spp. was studied by distinct authors at different times, including widow spiders in North [14-19] and South America [9, 12, 20-26]. In March 2021, the World Spider Catalog [27] listed 32 valid species for the genus *Latrodectus*. Whereas *L. geometricus* CL Koch, 1841 is considered semi cosmopolitan, another 18 species occur throughout different regions of the world: *L. cinctus* Blackwall, 1865; *L. dahli* Levi, 1959; *L. elegans* Thorell, 1898; *L. erythromelas* Schmidt & Klaas, 1991; *L. hasselti* Thorell, 1870; *L. hystrix* Simon, 1890; *L. indistinctus* O. Pickard-Cambridge, 1904; *L. karrooensis* Smithers, 1944; *L. katipo* Powell, 1871; *L. lilianae* Melic, 2000; *L. menavodi* Vinson, 1863; *L. obscurior* Dahl, 1902; *L. pallidus* O. Pickard-Cambridge, 1872; *L. revivensis* Shulov, 1948; *L. rhodesiensis* Mackay, 1972; *L. tredecimguttatus* (Rossi, 1790); *L. renivulvatus* Dahl, 1902; and *L. umbukwane* BMOG Wright, CD Wright, Lyle & Engelbrecht, 2019.

In the Americas, 14 of these species can be found: *L. geometricus* CL Koch, 1841; *L. antheratus* (Badcock, 1932); *L. apicalis* Butler, 1877; *L. bishopi* Kaston*,* 1938; *L. corallinus* Abalos, 1980; *L. curacaviensis* (Müller, 1776); *L. diaguita* Carcavallo, 1960; *L. hesperus* Chamberlin & Ivie, 1935; *L. mactans* (Fabricius, 1775); *L. mirabilis* (Holmberg, 1876), *L. quartus* Abalos, 1980; *L. thoracicus* Nicolet, 1849; *L. variegatus* Nicolet, 1849; and *L. variolus* Walckenaer, 1837. Due to frequent taxonomic changes of *Latrodectus* spp. over the years, in this review, we respected the specific nomenclature adopted in the original sources.

## Geographical distribution and epidemiology

The geographical and spatial distribution of *Latrodectus* spiders present great amplitude and variation [28-30]. Some species of this group have an almost exclusively endemic profile, such as *L. katipo* [31] and *L. variolus* [32]; other species have been introduced in two or more continents, like *L. hesperus* and *L. hasselti* [33-35]; there are also those with a highly invasive capacity, with semi-cosmopolitan distribution, such as *L. geometricus* [36-38]. This distribution is the result of the interaction of complex biological characteristics, including their reproductive efficacy associated with the invasion of new environments [39-43]; the development of semi-social behavior among members of certain populations [44-47]; dispersion models that allow a quick and widespread of spiderlings [48, 49]; tolerance and survivability within a wide thermal range [50, 51]; great variability in feeding habits [46, 52-54]; and the efficient plasticity of some species to environmental changes [15, 55-57]. This set of features results in the ability to infest and proliferate with great success even in environments strongly disturbed by humans [1, 37]. Therefore, the main barriers preventing the spread of terrestrial invertebrates [58] have little influence on the distribution limits of widow spiders, which find favorable conditions for survival and proliferation everywhere on the globe, except for Antarctica [31]. This phenomenon enhances contact with human populations and plays a great influence on the epidemiological profile of latrodectism [59-62].

**Figure 1:**
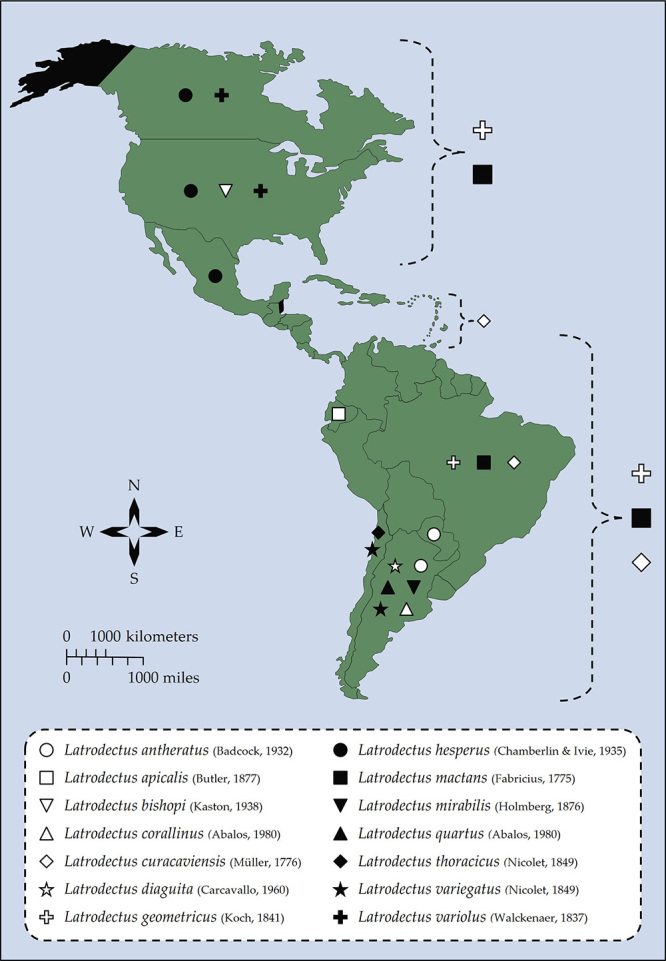
Geographical distribution of widow spiders in the Americas. The occurrence of fourteen *Latrodectus* species is currently recognized in the Americas. Symbols are placed on the countries where the presence of a given species has been reported. Brackets indicate an imprecise distribution across the continent. *Latrodectus geometricus* (white cross) has a wide distribution, occurring in many countries throughout the Americas. *Latrodectus mactans* (black square) is likely native to North America but has been introduced to South America. *Latrodectus curacaviensis* (white diamond) can be found in the Lesser Antilles and South America. Spiders from the *Latrodectus* genus have been reported in all the regions marked in green, although their identification at the species level is not always possible. No record of the presence of widow spiders is available for the regions marked in black (Belize and the state of Alaska, United States). This map is based on the list of *Latrodectus* species and their distribution available at The World Spider Catalog [27] as well as on the specialized literature on widow spiders, as mentioned before.

*Latrodectus* spiders have been reported in almost all countries in the Americas, as have the accidents caused by them. Their occurrence has been confirmed all over North America, including Canada [63], the United States [64, 65], Mexico [66], the Caribbean Islands [3, 67], and the Lesser Antilles [17]. Although data on latrodectism in Central America are scarce, the presence of widow spiders in this region has been reported by some authors; *Latrodectus* spp. are present in Guatemala [68], Honduras [69, 70], El Salvador [71], Nicaragua [72], Costa Rica [73], and Panama [74]. In South America, spiders from this genus can be found in Ecuador [3], Galapagos Islands [75], Bolivia [76], Peru [26, 76], Venezuela [26, 77], Colombia [26], Chile [26, 76, 78], Argentina [26, 76, 79], Brazil [1, 26, 76], Paraguay [26, 76], Uruguay [26, 76], the Guianas [26, 80], and Suriname [80]. Figure 1 shows the geographical distribution of *Latrodectus* species in the Americas.

Due to the wide distribution of these spiders, latrodectism is considered a semi-cosmopolitan health grievance. Human envenomation by *Latrodectus* spiders can occur either by contact with isolated specimens [81] or with dense population aggregates that some species form under favorable environmental conditions [31, 59, 60, 82]. These differences influence the dynamics of accidents caused by both invasive and endemic species, which result in peculiar clinical characteristics [31, 59, 82].

The real profile and epidemiological importance of *Latrodectus* spider bites are still unknown for some reasons: (1) it is often difficult to accurately diagnose these accidents; (2) the taxonomic identification of the etiologic agent is infrequent [78, 81, 83, 84]; (3) these spiders exhibit non-aggressive behavior [57]; (4) the lethality rate is low and many accidents result in mild clinical manifestations [1]; (5) many accidents remain unreported due to the practice of unsupervised home treatments [64, 65, 82]; and (6) accidents are underestimated in several countries, some of which lack an official reporting system [59].

Even without precise coverage, latrodectism is usually considered one of the worst and most serious cases of araneism in the world due to its dramatic and prolonged morbidity [85]. This motivates frequent discussions on isolated cases or few reports [63, 86], epidemiological analysis of limited chronological or geographical scope [87], and the dilution of the latrodectism understanding by its inclusion in general analyses of animal envenomation [82, 88].

Bettini [59] published one of the first and broadest compilations of the epidemiological knowledge available on latrodectism. His work was based on the review of a previous study on the cases that occurred in Italy between 1946 and 1951 and on the analysis of the available literature on latrodectism epidemic outbreaks. In an attempt to understand the epidemiology of venomous bites, he discusses the biology of spiders along with environmental and human factors that correlate to envenomation. The concepts introduced by Bettini [59] were adopted and widely used in many later studies on latrodectism. His observations and inferences have been discussed and corroborated by subsequent epidemiological and biological studies [2, 37, 88].

This study also introduced the idea of possible time cycles of latrodectism, consisting of "outbreaks epidemic" and "silence" periods. During the outbreaks, more medical and biological papers were published due to the sense of emergency generated by the frequency of spider bites. The subsequent periods of silence were marked by the absence of accidents and a proportional lack of information on the subject. These cycles would often have heterogeneous behaviors, lasting for periods as short as a few weeks or longer than a decade. This pattern, which is influenced by biological, environmental, and human conditions, contributes to the difficulty of establishing a robust epidemiological picture of latrodectism [3, 64, 82, 89].

Due to these difficulties, the vast majority of the available analyses focus primarily on pathophysiological manifestations and the evolution of clinical cases, as seen in studies from Australia (*L. hasselti*, 68 cases) [90], South Africa (*L. indistinctus* and *L. geometricus*, 45 cases) [86], Brazil (*Latrodectus* spp., 77 cases) [1], Spain (*L. tredecimguttatus*, 12 cases) [91], and the United States (*Latrodectus* spp., 163 cases) [64].

Bettini [59] has drawn attention to the heterogeneous geographical distribution of epidemic outbreaks of latrodectism, which have a certain level of “endemism”. This phenomenon is the result of many factors, such as population variations and biological requirements like temperature ranges [92], the substrate for web construction [93], types and abundance of prey [53, 94], and factors of general impact on the spiders’ ecology (infectious agents, parasites, predators, etc.) [95]. Other determinants for latrodectism identified by Bettini [59] are the density of human populations and the activities that can favor their contact with spiders. Most studies indicate latrodectism as a grievance that affects mainly the rural area, due to the positive relationship found between certain types of culture and the increase in populations of these spiders, especially in Europe [88, 89], South America [76], and Oceania [31]. However, some publications indicate the urbanization of these spiders and accidents in different countries [37, 50].

Some authors discuss the importance of phenology of *Latrodectus* spp. for the occurrence of accidents [46, 96]. In general, accidents tend to increase in the hottest months of the year in both tropical and temperate countries [1, 62]. However, species that colonize environments modified by humans find appropriate conditions during the whole year, so the frequency of accidents remains stable throughout the months. On the other hand, for the species found in crops, the planting and harvesting cycles play an important role in the seasonality of widow spider bites [65].

Epidemiological studies in the Americas are still scarce. Grisolia *et al*. [62] published an analysis about the accidents that occurred in the province of Buenos Aires (Argentina) between 1979 and 1988, with a total of 281 reported cases (an average of 28 accidents per year). Because the accidents were related to the rural environment and men were more likely to work on crops than women, males (80%) were more affected by the spider bites than females (20%). The number of cases was higher in the hottest months of the year (November to March), which differed from what was observed in the province of Santiago del Estero, in the north of Argentina, where the cases concentrated from March to May [97].

Schenone and Correa [51] performed an analysis of 150 cases of latrodectism that occurred during the summer between 1983 and 1984 in various regions of Chile. In this study, men aged 10 to 39 years were more affected by these accidents, which occurred mostly between 10 a.m. and 7 p.m.

In Brazil, the retrospective study by Lira-da-Silva *et al*. [1] points out the relevance of latrodectism in the city of Salvador (state of Bahia). The analysis of the accident reports from 1980 to 1990 revealed a total of 77 cases of latrodectism. The main species involved in the accidents was *L. curacaviensis*, which proliferates in ravines, coconut shells, crevices of stones, gardens, and interiors of homes. The majority (70%) of the victims were men between 10 and 29 years old. Importantly, this study shows that envenomed people who received specific antivenom had a shorter hospital stay than those who had not. Moreover, the accidents occurred predominantly in the urban area (57%), similarly to isolated *Latrodectus* sp. spider bites reported in other Brazilian states [98, 99].

## Clinical symptoms

Maretić [4] reported similarities in the clinical symptoms of the envenomation caused by distinct species of widow spiders, such as *L. mactans, L. hesperus, L. variolus*, and *L. bishop* (the Americas); *L. tredecimguttatus* (Eurasia); *L. cinctus, L. indistinctus*, and *L. menavodi* (Africa); *L. hasselti* (Oceania); and the semi-cosmopolitan *L. geometricus*. For this reason, in this review, we described the general clinical symptoms of latrodectism using reports from the American continent and comparing them, when necessary, with reports from elsewhere. 

The venom of *Latrodectus* spp. is a complex mixture of components, composed mostly of proteins and peptides. These components play several biological roles, such as paralyzing, immobilizing, killing, liquefying prey, and restricting competitors. Additionally, *Latrodectus* spp. hold toxins not only in their venom glands but also in other body parts (legs and abdomen), as well as in their eggs and spiderlings [100].

The most frequent symptom of *Latrodectus* bites is severe burning pain radiating from the inoculation site, and generalized muscle pain, due primarily to muscular spasm, which intensifies over time [1, 4, 64]. The neuromuscular manifestations, present in both experimental and human envenomation, are characterized by muscle cramps, involuntary contractions, and hypertonia, leading to stiffness in the abdomen and lower limbs. The facial manifestations, collectively called “*facies latrodectismica*”, include twisted facial muscles, blepharitis, rhinitis, cheilitis, and masseter trismus. Other symptoms include salivation, sweating, precordial oppression, anxiety, and mental excitation, which are responsible for the “*pavor mortis*” (fear of death) reported by the patients. Hypertension and renal alteration (oliguria) are also reported. Death is not common even without the use of serotherapy but may still occur, largely due to pulmonary edema and cardiac failure [101].

*Latrodectus* envenomation in humans can also result in hypertension and tachycardia. Although ECG changes may be present, damage to the myocardium is uncommon and cardiac manifestations are considered rare. In these uncommon cases, the concentration of troponin is increased, and echocardiographic changes are reported in all studies, but vary according to the report [61, 102-105]. Cardiogenic pulmonary edema that required mechanical ventilation has also been described [103]. Additionally, the venom of *L. dahli* from Iran causes myocytolysis, myocarditis, and coagulation necrosis of the heart under experimental conditions; the mechanisms of myocardial damage are nonetheless unclear [106]. The massive release of catecholamines is a direct toxic effect of the venom’s major component, α-latrotoxin (α-LTX), and hypersensitivity reactions have been suggested by some authors [5, 107, 108].

Rhabdomyolysis is another uncommon condition that can be observed from *Latrodectus* bites [102, 107]. It may be a consequence of generalized hypercontraction and intense muscle cramps that are typical of this envenomation; as a result, renal damage (oliguria and anuria) can occur. This lesion may also be caused by fluid loss (sweating, vomiting, or sialorrhea), or even by the direct action of the venom that causes paresis upon sphincter or hypercontraction at the abdominal musculature [4]. 

**Figure 2:**
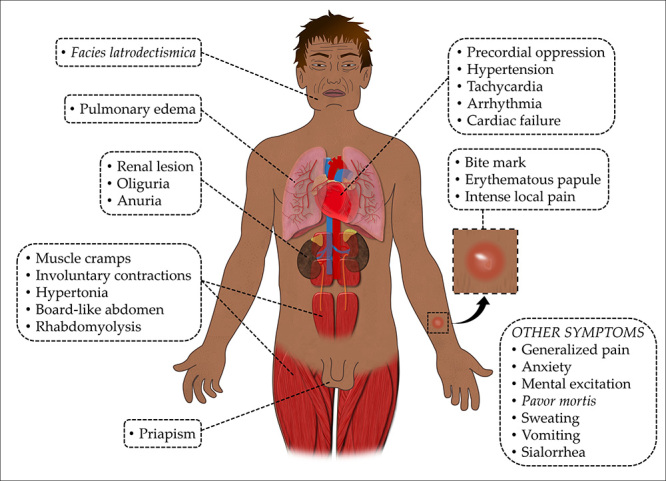
Local and systemic clinical manifestations of latrodectism.

A few reports have described priapism caused by *Latrodectus* spp. bites [109-112]. Some authors have suggested that the mechanism involved in venom-induced priapism is probably of the high flow type. In this case, there is no ischemia since the arterial blood flow to the penis is not compromised; therefore, the priapism is not very painful and can even be painless [109]. They suggest that the exhaustion of the noradrenaline after its intense release compromises penile detumescence. Additionally, the increase in acetylcholine from cholinergic nerves causes smooth muscle relaxation and the synthesis of nitric oxide from non-adrenergic and non-cholinergic nerves that promote vasodilation [102, 111]. Figure 2 summarizes the symptoms of *Latrodectus* envenomation.

### Differential diagnosis

Understandably, for a correct management of the envenomed patient, some conditions that can be mistaken with *Latrodectus* bites must be recognized for a differential diagnosis. Considering their neuromuscular manifestations, the differential diagnoses include alcohol or opiate abuse, organophosphate or strychnine poisoning, tetanus, rabies, renal colic, acute abdomen conditions, appendicitis, and peritonitis. Considering the cardiovascular manifestations, latrodectism can closely mimic acute myocardial ischemia [61, 113-115]. Some authors occasionally suggest other conditions: acute intermittent porphyria, inorganic lead intoxication (Saturnine colic) [61], and food poisoning [115]. Envenomation by other animals should also be considered, especially those that cause severe pain as a major symptom. Lastly, it is also important to consider skin and soft tissue infections, allergic reactions, dermatoses, and other skin-related issues that can be misdiagnosed as widow spider bites [116].

## Treatment

Several methods have been proposed for treating widow spider bites in the past. Most of these treatments tried to promote pain relief. Ancient therapies include the use of alcoholic beverages, hot baths, dancing, and even cocaine [117, 118]. However, even modern treatments are uncertain and variable [65], consisting mostly of muscle relaxants, opioids, benzodiazepines, calcium gluconate, and nonsteroidal anti-inflammatory drugs [64, 65, 119]. The efficacy of some of these treatments is nonetheless questionable, as shown by retrospective studies. In the case series by Clark *et al*. [64], 96% of the patients who received calcium gluconate, which has been extensively used since the mid-20^th^ century, experienced no pain relief nor resolution of other symptoms; these patients required a subsequent administration of opioids. Moreover, the use of opioids seemed to be effective in some cases, whereas other patients were unresponsive to this treatment. Only 55% of the bitten patients had satisfying levels of analgesia with the use of opioids; this rate increased to 70% when opioids were associated with benzodiazepines [64]. On the other hand, Monte *et al*. [65] have found a correlation between the use of benzodiazepines and symptoms enduring longer than 24 hours.

Unlike other non-specific therapies, *Latrodectus* antivenom promotes a quick resolution of the envenomation symptoms with a 100% success rate [64, 65, 119]. The serotherapy is associated with effective pain relief [65, 119, 120] and a reduction in hospitalization time [64]. Additionally, antivenom is a cheap treatment, with the cost of one vial estimated to be U$ 33.25 in 2012 [119]. The standard dose for treating widow spider bites is one vial of antivenom, although some patients might require a second vial for a full resolution of symptoms [64, 120]. Despite the effectiveness of antivenom, its use has been discouraged by many health practitioners due to the fear of anaphylactic reactions [65, 118, 121, 122].

In retrospective studies performed in the United States, the rate of antivenom use in patients bitten by widow spiders is around 2.2 to 3.8% [65, 122]. The concern regarding antivenom’s safety increased after the first report of death following the use of *Latrodectus* antivenom in the United States by Clark *et al*. [64]. A second fatality was reported almost two decades later by Murphy *et al*. [122]. Importantly, both patients had asthma, which has been pointed out as a contraindication for antivenom [122]. The circumstances of these fatalities should be considered before discrediting the safety of *Latrodectus* antivenom. Moreover, most of the data used to determine the risk of *Latrodectus* antivenom is extrapolated from the experience with snake antivenom, which overestimates the chances of anaphylactic reaction because the anti-*Latrodectus* serum is given in a much smaller volume [122].

Many authors agree that antivenom is a safe and effective therapy that should be used in severe *Latrodectus* envenomations, although some still advise that antivenom must be used with caution and only when every other therapeutic approach has failed [64, 119, 120]. If an anaphylactic reaction does occur, the antivenom infusion must be stopped as quickly as possible and the patient must be given support therapy with antihistamines, steroids, and epinephrine [122]. When patients are not eligible for serotherapy, opioids associated with muscle relaxants are recommended [118].

Some methods have been developed aiming to have a better safety profile of antivenom. The precipitation of inactive proteins and the enzymatic cleavage of antibodies into F(ab) or F(ab)_2_ fragments are some strategies used to reduce the immunogenicity of horse serum-based products [120]. As new technologies arise, horse serum may eventually not be needed whatsoever. For instance, the IgY-technology is a promising new method based on the production of specific antibodies from egg yolks. One of its advantages is the low immunogenicity to mammals, making it a potential substitute for the current method [123].

Cross-reactivity studies have shown that *Latrodectus* antivenom is not species-specific, suggesting that any anti-*Latrodectus* serum could potentially be used to treat envenomations caused by any *Latrodectus* sp. [118, 121, 124] and other Theridiidae spiders such as *Steatoda grossa* [125], which also has *Latrodectus*-like toxins, as discussed in further detail in the section “venom components”. Consequently, the identification of the species is not clinically relevant, as all patients will respond well to antivenom. Thus, it would be interesting from the perspective of public health to discuss the production of antivenom that could be distributed and used anywhere in the Americas regardless of the species used for production.

The idea of an anti-*Latrodectus* serum dates to the early 1900s, when the blood of a patient who had recovered from a widow spider bite was given intramuscularly to another bitten patient, who then showed a significant improvement [117]. In 1936, Mulford Biological Laboratories of Sharp and Dohme, in the United States, produced the first commercially available *Latrodectus* horse serum-based antivenom [118]. Eventually, other companies from different countries made *Latrodectus* antivenom available, as shown in Table 1. It is worth mentioning that the Instituto Vital Brazil (Niterói, Brazil) has already registered an anti-*Latrodectus* serum produced against *L. curacaviensis* venom [126, 127], which could potentially be used in the near future to treat the accidents caused by widow spiders in the Brazilian territory.

**Table 1:** List of the anti-*Latrodectus* antivenoms produced in the world.

Continent	Country	Producer	Species^*^
Oceania	Australia	Commonwealth Serum Laboratories (CSL)	*L. hasselti*
Africa	South Africa	National Health Laboratory Services (NHLS)	*L. indistinctus*
America	Argentina	Instituto Nacional de Producción de Biológicos (INPB)	*L. variegatus,*
			*L. antheratus,*
			*L. diaguita,*
			*L. corallinus,*
			*L. mirabilis,*
			*L. quartus*
	Brazil	Instituto Vital Brazil (IVB)	*L. curacaviensis*
	Mexico	Instituto Bioclon Laboratorio Silanes (BIOCLON)	*L. mactans*
	United States	Merck Sharp and Dohme International	*L. mactans*

^*^This table considers only the licensed antivenoms and the species listed herein are indicated in the package insert or on the website of the producers.

## Methods for venom collection

The small size of *Latrodectus* spiders resulted in the development of different techniques to obtain the small volume of venom they produce. The amount, composition, and properties of the venom obtained vary according to the extraction method. The most used techniques are crushing and washing the spider's cephalothorax followed by recovering the diluted venom; collecting venom in capillary tubes inserted in the glands’ lumen; surgically extracting the glands and homogenizing them in different buffers [128, 129]; electrically stimulating live specimens forcing venom ejection [130]; inducing spider bites on cotton or absorbent paper [131]; and stressing the specimens until they eject venom drops in capillary tubes [132]. Each of these techniques has its limitations such as the wide variation in individual responses of the extracted specimens; contamination by tissues or other body secretions; contamination by external agents; very low venom yield; and losses between processing steps [133].

## Experimental studies on *Latrodectus* venom

The difficulties regarding *Latrodectus* genus taxonomy, obtaining a reasonable amount of venom, and the limited number of research groups that have systematically studied these spiders are limitations for understanding the characteristics of their venom and the effects of envenomation. There have been only a few studies regarding the effects of crude venom from widow spiders of the American continent, in isolated systems. Some of these studies are reported herein.

### Toxicity

*Latrodectus* venom is widely known for its extreme toxicity. However, the LD_50_ data available in the literature are variable, considering the method of venom extraction, the test animals used, and the route of administration. Also, *Latrodectus* species have taxonomic inaccuracies that can compromise information.

McCrone [17] has found the following LD_50_ values after the administration of venom from *Latrodectus* spp. from the United States in mice by intraperitoneal route - *L. mactans mactans*: 1.3 (1.2 - 2.7) mg/kg; *L. variolus*: 1.8 (1.2 - 2.7) mg/kg; and *L. bishop*: 2.2 (1.29 - 3.74) mg/kg. De Roodt *et al*. [101], also using the intraperitoneal route in mice, has discovered a different range of LD_50_ values for spiders from different regions of Argentina - *L. mirabilis*: 0.155 to 0.6 mg/kg; *L. diaguita*: 0.31 to 1.08 mg/kg; and *L. corallinus*: 0.5 mg/kg^1^.

Different populations of *L. geometricus*, which generally causes only mild envenomation in humans, have been reported to have different LD_50_ values in experiments performed on mice: 0.43 mg/kg for spiders from Florida (United States) [17] and 0.225 mg/kg for spiders from Miranda (Venezuela) [134]. It is known that geographical differences can contribute to the intraspecific variability of venoms and their toxicity [135]; although there are no studies on the subject for *Latrodectus* spp., it is possible that variances in venom composition driven by different environmental pressures may have contributed to the discrepancy between the studies.

### Cell culture experiments

Using neuronal hippocampal culture and human embryonic kidney cells (HEK 293 cells), Parodi and Romero [136] proposed that the *Latrodectus* venom from Chile closes potassium channels, extending the action potential similarly to tetraethylammonium (a non-selective potassium channel blocker). These changes induce a spontaneous synaptic activity in a concentration- and time-dependent manner. The effect is reversible after removing the venom; moreover, it is abolished by heating the venom at 96°C for 45 min, indicating its thermolability.

### Cardiovascular system and muscle tissue

Romero *et al*. [137, 138] showed positive inotropic and chronotropic effects of the extract of *L. mactan*s venom glands from Chile on the cardiac papillary muscle of rats, similarly to sympathomimetics molecules. The authors suggested that the venom induces sympathetic tonus, which could explain the vascular and cardiac symptoms of the envenomation.

In some models of isolated smooth muscle, the venom induces a sustained tonic effect related to the permeability to Na^+^ and Ca^2+^ ions that modulate the contractile response. The response is characterized by a fast-phasic component followed by a slower and more sustained tonic component probably due to the release of adrenergic and cholinergic neurotransmitters [139]. However, other studies using the smooth muscle of the urethra and esophagus have shown that α-LTX can also release non-adrenergic and non-cholinergic mediators, particularly nitric oxide and intestinal vasoactive peptide [140, 141], thereby causing relaxation.

The ultrastructural analysis of skeletal muscle injuries caused by *L. geometricus* venom demonstrated the presence of eosinophil infiltration, edema of the sarcotubular systems, and rupture of cell membranes. The mitochondria and nuclei were degenerate and surrounded by necrotic areas and myofibrillar disorganization [134]. The mechanisms involved in this injury have not been investigated.

### Liver and kidney

The experimental injection of *L. dahli* venom in rabbits (0.5 mg/kg by subcutaneous route) induced an increase in the levels of alanine aminotransferase, aspartate aminotransferase, alkaline phosphatase, and bilirubin, suggesting liver damage [142]. Hepatic damage had already been reported with the presence of liver edema, massive hyperemia, and lobular necrosis [143, 144]. Moreover, the increased levels of creatinine, albumin, and urea suggest the impairment of kidney function [142]. Maretić and Stani [145] have suggested that the kidney damage caused by *Latrodectus* envenomation is secondary to intense dehydration due to sweating and sialorrhea.

### Pain and inflammation

Although the main symptom of latrodectism is pain, only recently Lauria *et al*. [146] described the mechanisms involved in the nociception induced by *L. curacaviensis* venom from Rio de Janeiro (Brazil). This venom causes intense and heat-sensitive spontaneous nociception, mediated by serotonin and bradykinin receptors, and TRPV1 channels. It has been suggested that the presence of serotonin in spider venoms can be associated with pain production, self-protection, and the facilitation of venom uptake by increasing local blood flow and cell permeability [147]. *L. curacaviensis* venom also induces mechanical allodynia, which is reduced by the pharmacological inhibition of H_1_ histamine receptors and TRPV1 channels. Additionally, the nociceptive effect of the venom is abolished when it is heated to 90°C for 10 min, indicating the thermolability of the nociceptive toxins [146].

The inflammatory profile of *Latrodectus* venoms has also been poorly investigated. *L. curacaviensis* venom induces paw edema of low intensity and short duration in mice, mast cell degranulation, and the increase in local IL-1β levels [146]. Liver and myocardium edema have also been observed after the inoculation of *L. dahli* venom in rabbits [106].

## Venom components

Despite their vast geographical distribution and variety of species, all spiders from the *Latrodectus* genus express proteins of high molecular weight that are neurotoxic to vertebrates (α-LTX), insects (latroinsectotoxin; LIT), and crustaceans (latrocrustatoxin; LCT). These proteins have acid isoelectric points, ranging from pH 5.2 to 6.38. Since these molecules share similar features and mechanisms of action, suggesting that they are related, scientists decided to group them in a family named latrotoxins [148]. It is noteworthy that most of the information about the genus was acquired using the venoms from *L. m. tredecimguttatus* and *L. tredecimguttatus.*

Longenecker *et al*. [149] observed that the aqueous components of venom glands macerated (ACVGM) from *L. m. tredecimguttatus* caused the depletion of synaptic vesicles at the neuromuscular junction (NMJ) of frogs. Frontali *et al*. [150] were the first to isolate and characterize the B5 fraction, which contained a 130 kDa protein that was responsible for the neurotoxicity of the venom upon vertebrates. Tzeng and Siekevitz [151] improved the purification steps and named this protein α-LTX since it was the first characterized toxin of this genus. The toxin is a homodimer with three specific regions (wing, body, and head) that assumes a tetrameric form in the presence of divalent cations, becoming active. The oligomer resembles a four-bladed propeller with a channel in the center [152, 153].

Some of the characteristics of α-LTX include the ability to fuse into lipid bilayers [154]; the evocation of a substantial and fast influx of Ca^2+^ ions into different cells, promoting the release of neurotransmitters such as noradrenaline, dopamine, acetylcholine (Ach), and γ-aminobutyric acid (GABA) [155]; the depletion of synaptic vesicles at the NMJ [156], on slices of the cerebral cortex of mice [151, 157], and from rat synaptosomes preparations [156]. The toxin also induces the exocytosis of different hormones: catecholamines by chromaffin cells [158]; insulin by pancreatic ß-cells [159]; and antidiuretic hormone and oxytocin by the neurohypophysis [160], but this process depends on Ca^2+^ [161-163]. Hence, it is possible to assume that all secretory cells are sensitive to the action of α-LTX since the molecule was able to induce the secretion of hormones and several neurotransmitters.

**Figure 3:**
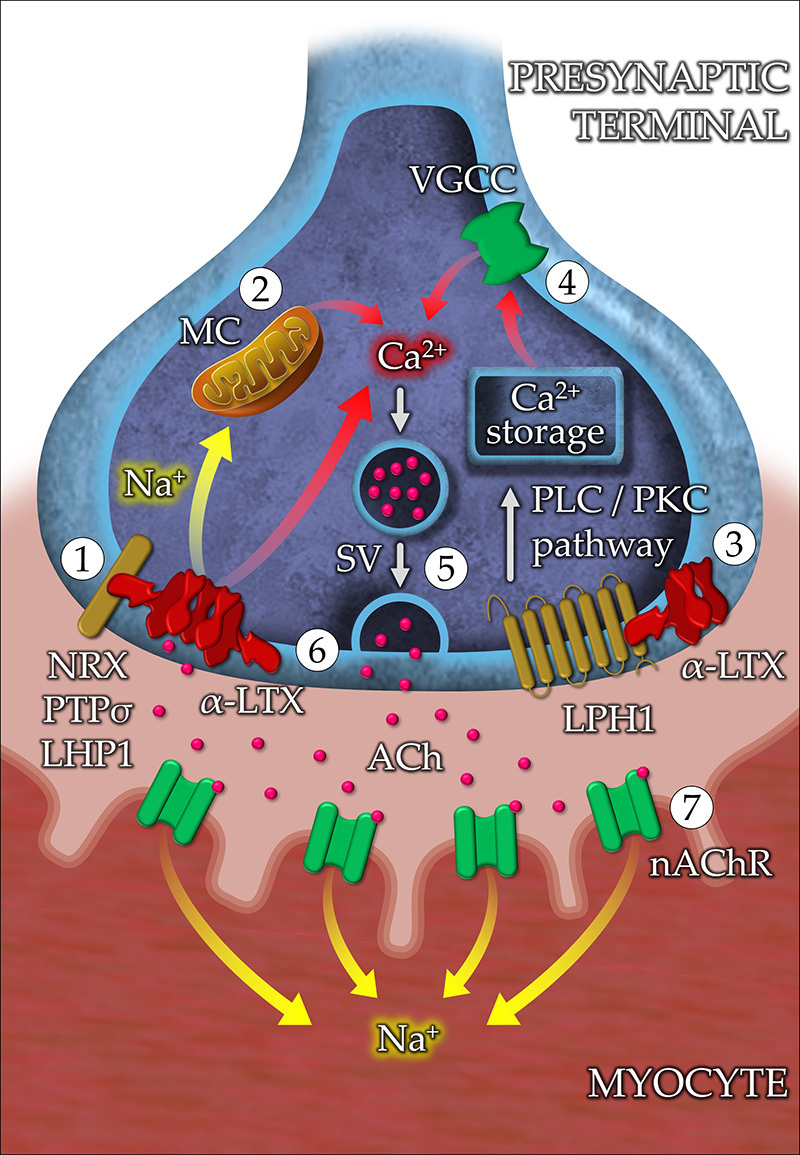
Proposed mechanisms of neurotoxicity of α-LTX in the neuromuscular junction. **(1)** In the presynaptic membrane, the wing region (N-terminal domain) of α-latrotoxin (α-LTX) interacts with neurexin-Iα (NXR-Iα), protein tyrosine phosphatase σ (PTPσ), and latrophilin 1 (LPH1). This interaction facilitates the insertion of the tetramer into the lipid bilayer, allowing the influx of monovalent (Li^+^, Cs^+^, Na^+^, K^+^) and divalent (Ca^2+^, Ba^2+^, Mg^2+^) alkali cations. **(2)** The excess of Na^+^ induces the release of more Ca^2+^ from the mitochondria into the cytoplasm. **(3)** α-LTX triggers LPH1 signaling, resulting in the activation of the phospholipase C (PLC)/protein kinase C (PKC) pathway. PLC increases the levels of inositol trisphosphate, promoting the release of intracellular Ca^2+^ stored in the endoplasmic reticulum. **(4)** The depolarization of the presynaptic terminal activates voltage-gated calcium channels (VGCC), allowing the influx of more Ca^2+^. **(5)** The massive increase in intracellular Ca^2+^ promotes the mobilization of synaptic vessels (SV) containing the neurotransmitter acetylcholine (Ach). **(6)** The release of Ach into the synaptic cleft can be either due to the fusion of SVs with the plasma membrane or due to the leakage of the neurotransmitter through the α-LTX pore. **(7)** The interaction of Ach with the nicotinic acetylcholine receptor (nAChR) present in the membrane of the myocyte causes depolarization and muscle contraction.

The mechanism of action of α-LTX is composed of complementary pathways: (1) the formation of a pore in the presynaptic membrane after the toxin insertion allowing cationic influx, and (2) the activation of intracellular signaling cascades that lead to a global increase in the amount of calcium (Figure 3). The interaction between the membrane receptors and α-LTX occurs through a Ca^2+^-dependent mechanism by neurexin-Iα (NXR-Iα) or Ca^2+^-independent mechanisms, by protein tyrosine phosphatase σ (PTPσ) and latrophilin 1 (LPH1), also named calcium-independent receptor of α-LTX (CIRL). The receptors interact with the toxin's upper portion, in its tetrameric form, while the base inserts itself in the membrane, permeating the lipid bilayer. These interactions facilitate the insertion of the toxin into the lipid bilayer and therefore pore formation. Additionally, the interaction between the toxin and LPH1, a G protein-coupled receptor, activates the protein kinase C, followed by the release of calcium from the endoplasmic reticulum [152, 153, 162, 164-168]. In summary, α-LTX interacts with specific receptors from neuronal and neuroendocrine terminals, resulting in a massive release of neurotransmitters based on pore formation and intracellular signaling cascades.

Frontali *et al*. [150] also demonstrated the toxicity of fraction C from ACVGM to insects, since it killed flies and blocked the heartbeat of cockroaches. Following on, Kovalevskaia *et al*. [169] isolated and identified a 120 kDa protein responsible for this effect and labeled it as α-latroinsectotoxin (α-LIT). This toxin displays specific characteristics: (1) the binding and fusion to membrane preparations, in a similar manner to α-LTX, but specific to insects [170]; (2) the increase of the frequency of glutamatergic miniature end-plate potentials at NMJs from insects, followed by a blockade of the synaptic transmission; (3) an absence of potentiation or antagonist action if associate to α-LTX; and (4) it is not recognized by polyclonal antibodies against α-LTX [169, 171, 172].

In addition to α-LIT, four other LIT subtypes were isolated: β-LIT (140 kDa), γ-LIT (120 kDa), ε-LIT (110 kDa), and δ-LIT (110 kDa). All subtypes were toxic to wax moths, with α-LIT being the most lethal molecule [148, 173]. Among the four subtypes, only δ-LIT had its primary sequence reported, which was shorter than those of α-LTX and α-LIT. This molecule also acts similarly to α-LIT [174].

According to Frontali *et al*. [150], fraction E from ACVGM is responsible for the depolarization of stretch receptors of crayfish. Fritz *et al*. [175] observed the specificity of this fraction against crustaceans by reporting the release of neurotransmitters in NMJs from lobsters, among other models. Grishin [148] isolated a 120 kDa protein responsible for these effects and proposed the name of α-latrocrustatoxin (α-LCT).

The paralogs α-LTX, α-LIT, δ-LIT, and α-LCT from *L. m. tredecimguttatus* venom are long polypeptides (1,200 to 1,400 amino acid residues) that share 30-60% of identity [148, 174, 176, 177]. These molecules have common characteristics such as moderate levels of amino acid homology (17%); proteolytic processing of both N- and C-terminal regions, by furin-like proteases, as steps of the toxin’s maturation [170, 178, 179]; and comparable domain organization. The mature toxins are composed of an N-terminal domain (with similar features to all latrotoxins, indicating an ancestral gene) and a C-terminal part (formed by two-thirds of ankyrin-like sequences that range from 13-22 repeats) [148, 171, 172, 174, 180]. Researchers believe that the ankyrin repeats, known for coordinating the interaction of several proteins, could act in the high-affinity binding between these molecules and their receptors, even though the receptors for LITs and LCT are unknown. The compilation of data about these toxins reveals a similar mechanism across phyla [172].

Other molecules described in the venoms of *Latrodectus* spp. are the Low Molecular Weight Proteins, also called latrodectins [181]. Latrodectin-1 (approximately 8,0 kDa) and latrodectin-2 (9,5 kDa) are acid proteins of unknown function that share structural homology and co-purify with α-LTX and LITs, respectively [181-183]. Studies demonstrate that latrodectins were derived molecules from the Crustacean Hyperglycaemic Hormone / Ion Transport Peptides superfamily that suffered positive and negative selections [184, 185]. Latrodectin-1 is present in the venom in equal amounts to α-LTX; it can be found free or forming a complex with α-LTX [183]. Studies revealed that a specific antibody against this molecule inhibited the effects triggered by α-LTX [186]. McCowan and Garb [185] proposed that latrodectin-1 could modulate the transport of calcium ions around or near nerve cells, thereby enhancing the toxicity of α-LTX. Other possible mechanisms of latrodectin-1 are stabilizing α-LTX and/or promoting its connection to the plasma membrane, facilitating the pore formation. Both hypotheses, however, have not yet been validated [162].

Some components from *Latrodectus* spp. venoms have been isolated or biochemically tested for their activity. In addition to latrotoxins and latrodectins, previous studies reported the presence of a variety of molecules, such as protease inhibitors and different types of enzymes (proteases and hyaluronidase) [187], and other components as part of the venom such as adenosine, guanosine, inosine, and serotonin [113, 147, 187], shown in Table 2.

**Table 2:** List of isolated or biochemically tested components (enzymes or inhibitors) from the venoms of *Latrodectus* spp.

Molecules	Species
α-LTXs	*L. m. tredecimguttatus*/*L. tredecimguttatus*: α-LTX-Lt1a [176]
	*L. mactans*: α-LTX-Lm1a [188]
	*L. hesperus*: α-LTX-Lhe1a [188]
	*L. hasselti*: α-LTX-Lh1a [188]
LITs	*L. m. tredecimguttatus*/*L. tredecimguttatus*: α-LIT-Lt1a [177],
	δ-LIT-Lt1a [174], ß-LIT, γ-LIT, and ε-LIT [148]
	*L. hasselti*: α-LIT-Lh1a and δ-LIT-Lh1a [188]
	*L. hesperus*: δ-LIT-Lhe1a [188]
α-LCT	*L. m. tredecimguttatus*/*L. tredecimguttatus*: α-LCT-Lt1a [148]
Latrodectins	*L. m. tredecimguttatus*/*L. tredecimguttatus* [182, 183]
	*L. geometricus* [185]
	*L. hesperus* [185]
Peptide inhibitor of angiotensin-converting enzyme	*L. tredecimguttatus* [147]
Hyaluronidase	*L. tredecimguttatus* [113]
	*L. mactans* [113]
Collagenase	*L. geometricus* [134]
Phosphodiesterase	*L. mactans* (from North America^*^) [189]
Bradykinin-inactivating enzyme (kininase)-thiol endopeptidase	*L. tredecimguttatus* [190]^**^
Serotonin	*L. tredecimguttatus* [191]
Free amino acids (glutamic acid, aspartic acid, GABA, and taurine)	*L. tredecimguttatus* [3, 147]
Purine derivatives (adenosine, guanosine, inosine, and 2,4,6-trihydroxypurine)	*L. menavodi* [192]

^*^Considering the great distribution and the taxonomic uncertainty of the species, the authors prefer to identify the origin of the venom when present in the original work.

^**^The authors reported that *L. tredecimguttatus* has no proteolytic activity against casein and hemoglobin. Moreover, Frontali *et al*. [150] did not detect lipolytic or proteolytic action of a toxic fraction of *Latrodectus* venom against Azocoll.

The studies of venom components from the American widow spiders are scarce and limited to some latrotoxin family members and latrodectins from *L. mactans*, *L. hesperus*, and *L. geometricus*. Graudins *et al*. [188] reported α-LTX, α-LIT, and δ-LIT molecules in the venom of the phylogenetically related spider *Steatoda grossa* (CL Coch, 1838) (Theridiidae). Additionally, Haney *et al*. [193] have described orthologous proteins from this family and other components in the venom glands from the genus *Latrodectus* and *Steatoda*. In the light of adaptive evolution, the authors reported that switches in the expression of these genes from other tissues to the venom gland associated with positive selection and gene duplication contribute to the diversity of molecules. Such evolutionary process could lead to variations in venoms’ toxicity. More recently, Dunbar *et al*. [194] have shown that *Lactrodectus*-like toxins are also present in the venom of *Steatoda nobilis* (Thorel, 1875), suggesting that the presence of these molecules among Theridiidae spiders could be more common than initially thought. Graudins *et al*. [188] have confirmed that α-LTX from *L. mactans, L. hesperus*, and *L. hasselti* elicited neurotransmitter release in the same manner as α-LTX from *L. tredecimguttatus*. However, the monoclonal antibody (4C4.1) against α-LTX from *L. tredecimguttatus* was unable to neutralize their toxicities. They deduced the sequence of α-LTX from *L. hasselti* by tracking the similarities regarding the number of amino acid residues, domain organizations, and proteolytic processing with α-LTX from *L. tredecimguttatus*. The sequences presented high identity (93%) with the presence of non-conservative substitutions at specific residues that precluded the neutralization by 4C4.1.

In another study, Parodi and Romero [136] reported the absence of α-LTX in *L. mactans* from Chile. The authors suggested that the latrodectins are responsible for the toxic effects observed in envenomation by this species. However, in a broader analysis with sequences of α-LTX from members of Theridiidae (*Parasteatoda* Archer, 1946; *Steatoda*; and *Latrodectus*), Garb and Hayashi [195] demonstrated the presence of α-LTX in venom samples from Chile and an identity of 94% among several *Latrodectus* species. They also declared that the occurrence of positive and negative selections in the gene α-LTX could explain the higher toxicity of this molecule in the *Latrodectus* genus. These findings confirm the existence of α-LTX homologs among species from the Theridiidae family. It also clarifies the cross-reactivity observed in antivenom studies [196]. McCowan and Garb [185] described latrodectins’ paralogs in the venoms of *L. hesperus* and *L. geometricus*, discussing the higher similarity of these toxins within the *mactans* clade and therefore the closer phylogenetic relationship between *L. hesperus* and *L. tredecimguttatus* when compared to *L. geometricus* (*geometricus* clade).

## Proteome and transcriptome analyses of *Latrodectus* venom

Even though widow spiders are medically relevant, the number of studies that investigate and characterize the molecules of their venoms is scarce, compromising the understanding of all mechanisms involved in the envenomation. The implementation of high-throughput methods such as transcriptomic and proteomic was a breakthrough step in the development of studies related to venom. There are currently six studies that characterize the venom and/or venom glands using proteomic and/or transcriptomic approaches. Two of them describe the venoms from two American species (*L. hesperus* and *L. mactans*), whereas one reported the venom components of the semi-cosmopolitan species *L. geometricus*. These data reinforce the impressive lack of information about the venoms from American widow spiders.

In recent years, high-throughput methods have gained notoriety in the study of widow spiders due to the limitations regarding the amount and purity of the venom that can be obtained from widow spiders, as discussed in the section “methods for venom collection”. Researchers have employed this approach to different biological materials, amounting to ten studies to gather more information about this genus. These studies encompass eggs [197, 198], silk glands [199-204], newly hatched spiderlings [205], and different tissues from adult spiders [206]. Notably, the use of genome and/or transcriptome techniques make it easier to define the protein content of these biological tissues. Here we discuss the results of the analyses of the venom or venom glands. Table 3 presents a compilation of the different studies conducted to date using transcriptomics and proteomics for the analysis of *Latrodectus* spp.

**Table 3:** Compilation of the studies on *Latrodectus* spiders using proteomics and transcriptomics approaches.

Species	Origin	Analyzed material	P/T
*L. tredecimguttatus*	Not informed	Eggs [198]	P
	Not informed	Eggs [197]	T
	China	Newly-hatched spiderlings and adult spiders [205]	P
	Not informed	Venom glands (F) [207]	T
	China	Venom extracted from dissected venom glands (F) [129]	P
	China	Venom extracted by ES (F) [208]	P
*L. hesperus*	Not informed	Silk glands (M and F) [199]	T
	United States	Silk glands (F) [200]	P
	Database	Different tissue types [206]	T
	Database	Different tissue types [201]	T
	Database	Different tissue types [202]	T
	United States	Different tissue types (F) [203]	T
	United States	Venom glands, silk glands, and cephalothorax (F) [204]	T
	Not informed	Silk glands, cephalothorax minus venom glands, venom glands, and venom extracted by ES (F) [209]	P/T
*L. geometricu*s	United States	Different tissue types (F) [203]	T
	Not informed	Silk glands (M and F) [199]	T
	Thailand	Venom extracted by the cold shock method (F) [210]	P
*L. mactans*	Alphabiotoxine Laboratory	Venom glands and venom extracted by ES [211]	P/T

P: proteome analysis; T: transcriptome analysis; M: male; F: female; ES: electrical stimulation.

Duan and coworkers [129, 208] conducted the initial proteomics studies using the venom from *L. tredecimguttatus*. In the first work, the authors obtained the sample from dissected venom glands punctured with a needle [129], whereas in the second work, the authors used electrostimulation [208]. These strategies allowed the identification of 86 and 122 molecules in each study, respectively. The authors classified these molecules according to their biological functions. The main classes were latrotoxins (α-LTX, α- and δ-LIT, and α-LCT precursors), hydrolases, other enzymes, and proteins, some of which have an unknown function. Each study, however, presented exclusive categories. In the first work, these molecules were in the cytoskeletal and related proteins class; the researchers reported the presence of contaminants, such as histiocytic proteins [129]. In the second study, the authors have described binding proteins and other specific molecules (leech-derived tryptase inhibitor trypsin complex, venom allergen antigen 5-like protein, and fucolectin) [208]. Recently, Khamtorn *et al*. [210] reported a partial proteome profile of *L. geometricus* venom using a cold shock method during the collection step. The authors described, similarly to Duan *et al*. [129, 208], the presence of members from the latrotoxin family, hemocyanins, proteases, and other enzymes among the venom components.

Data generated from transcriptome analyses partially overcome the issue of obtaining venom in high amounts, for it allows the large-scale production of specific toxins in gene expression systems. There are currently only three transcriptome studies using venom glands from the *Latrodectus* genus, and all applied next-generation sequencing, followed by de novo assembly. The pioneers were He *et al*. [207], who searched for novel toxins from *L. tredecimguttatus*, enabling the identification of 146 toxin-like proteins sequences grouped in the following categories: (1) neurotoxins (members of α-LTX-Lt1a family 1, α-LTX-Lt1a family 2, α-LIT-Lt1a, δ-LIT-Lt1a, ankyrin family, sperm-coating glycoprotein, and lycotoxin families); (2) assistant toxins (theriditoxin family); (3) proteases (trypsin family); (4) protease inhibitors (ctenitoxin family); and (5) toxins with unknown functions (scorpion toxin-like and orphan families). In a multi-tissue analysis of *L. hesperus*, Haney *et al*. [209] compared selected venom gland-specific transcripts with the venom. This strategy allowed the discovery of 62 transcripts sequences with homology to known proteins and the identification of 61 proteins; the molecules identified were classified as toxins or enzymes. In the first group of molecules, there were latrotoxins, latrodectins, inhibitor cystine knots (ICK) toxins, cysteine-rich secretory proteins (CRISPs), and leucine-rich repeats (LRR) proteins. In the second group of molecules, there were metalloproteases, serine proteases, chitinases, and hyaluronidases. Oldrati *et al*. [211] compared the transcripts from venom glands and the venoms of phylogenetically distant species: *L. mactans* and another three species belonging to different genera. The authors focused the analysis on a subgroup of molecules, the cysteine-rich peptide toxins, differing from the previous studies.

Despite their distinct origins, there is evident conservation of the molecules present in the venom glands of *L. tredecimguttatus* (Asia) and *L. hesperus* (America). The presence of these components is related to their similar envenomation symptoms and could be partially explained by the high identity among the α-LTXs sequences. In general, proteins and peptides with homology to known toxins were present in the venoms. Latrotoxins (ankyrin superfamily), latrodectins (theriditoxin members), ctenitoxins, and CRISPs were the toxins or toxin families found. Additionally, metalloproteinases, hyaluronidases, and a serine protease were also present in both venoms. However, some differences were also observed between them, for instance, the venom glands of *L. tredecimguttatus* contain molecules with a characteristic pattern of six highly conserved cysteines, classified as members of the scorpion toxin-like family [207]. Moreover, LRR proteins and chitinases were exclusively found in the venom of *L. hesperus* by Haney *et al*. [209]. Besides, although both species present latrotoxins, these molecules, and their motifs diverge phylogenetically [209, 212]. Haney *et al*. [209] reported more than 20 divergent latrotoxins paralogs, expressed in the venom glands of *L. hesperus* (the Americas), one of which had already been described by Graudins *et al*. [188]. They also identified orthologs of α-LTX, but the biological action of these molecules still needs to be tested. Whether these differences are relevant in the distinction among them (Old World vs. New World) is yet to be confirmed.

Haney *et al*. [209] analyzed the whole venom from *L. hesperus*, whereas Oldrati *et al*. [211] focused on the cysteine-rich peptide toxins from *L. mactans*. Both researchers used species found in the Americas. The authors compared the venom components (obtained by electrostimulation) with the sequences found in the transcriptome of the venom glands in an attempt to pinpoint the secreted molecules. Among these molecules, Haney *et al*. [209] identified latrotoxins, latrodectins, LRR proteins, ICK toxins, putative ICKs, CRISPs, and several enzymes (metalloproteases, serine proteases, hyaluronidases, and chitinase). Oldrati *et al*. [211] identified peptides from three distinct families as *Cupiennius salei* (Keyserlingi, 1877) toxins (CSTX), peptidase S1, and arthropod CHH/MIH/GIH/VIH hormone. In both studies, the researchers noticed the CSTX molecules, classified as ICK toxins, in the analysis of *L. hesperus* venom.

Curiously, the only similarity among the proteomic studies on the venoms of *L. tredecimguttatus* [129, 208], *L. hesperus* [209], and *L. geometricus* [210] is the presence of latrotoxins, latrodectins, and some enzymes. We believe that some of those differences are related to the partial proteomic profile obtained by Khamtorn *et al*. [210] and the different databases used in the *L. tredecimguttatus* and *L. hesperus* studies. Another difference lies in the parameters used to classify the molecules as toxins. Duan *et al*. [129, 208] loosely classified their molecules using generic biological functions. Haney *et al*. [209] used a set of defined parameters such as the presence of signal peptide and conserved domains in the sequences and the prediction of potential toxicity for the molecules; the use of these parameters was only possible due to the gain of information provided by the transcriptome analysis. No transcriptome nor genome data were available at the time Duan *et al*. [129, 208] performed their work.

The reasons why the envenomation varies remain unexplained. The interspecific differences of the *Latrodectus* genus could explain some of these variations. Even if α-LTX sequences present a high identity among *Latrodectus* spp., the variability (5.8%) within the *mactans* clade cannot be ignored [188, 195]. Also, the actions of the remaining components of the venom should not be disregarded since they lack characterization. Therefore, combining analyses of transcriptomic and proteomic data and comparing them among species is essential. Ultimately, the association of these techniques will allow a better understanding of the complexity of the venom and the mechanisms involved in the envenomation.

## Beyond biological and biochemical characterization: biotechnology opportunities

The molecules from the venom and body parts of *Latrodectus* spp. have also been studied for their biotechnological potential and some patents have been applied/granted. For instance, a fragment of a peptide isolated from *L. mirabilis* from Chile has been proposed as a spermicide contraceptive drug [213]. Another example is the use of polypeptides derived from α-LTX in the treatment of erectile dysfunction [214]. However, the biotechnological uses are not restricted to these products and the possibilities are many, as shown by the studies from different fields described in the subsections below.

### Insecticidal activity

Because of their feeding habits, the venom of spiders contains toxins that directly affect insects; therefore, some of these molecules can potentially be used as insecticides. A clear example is the LITs from widow spiders, as proposed by Rohou *et al*. [172]. Toxins from this family act specifically toward insects, either by a pore-forming mechanism or by the interaction with membrane receptors leading to neurotransmitter release. Moreover, latrodectins have been shown to increase α-LTXs activity toward insects. Nevertheless, there are limitations to the biotechnological use of LITs. Firstly, their receptors are still unknown; and secondly, LITs have high molecular weight, so they are quickly cleaved and deactivated by insects’ proteases [215]. The insecticide molecules are not restricted to the venom; a component purified from *L. tredecimguttatus* eggs (latroeggtoxin-III, a 36 kDa protein) showed a potent toxicity on cockroaches [216].

### Antimicrobial activity

Widow spider-derived molecules have also been shown to display antimicrobial activity. Khamtorn *et al*. [210] have described the antibiotic action of *L. geometricus* venom against gram-positive bacteria. Makover *et al*. [217], also working with *L. geometricus*, have shown that components from both extract and surface of eggs play an important role in the protection of eggs against infection. Moreover, latroeggtoxin-IV, an egg toxin from *L. tredecimguttatus*, exerts a potent antibiotic activity against gram-positive and gram-negative bacteria; this effect is comparable to that from ampicillin, a broad-spectrum antibiotic [216]. These results altogether indicate the presence of molecules in *Latrodectus* spp. venom and eggs with potential application as antibiotics.

### Antitumor activity

A few articles analyzed the antitumor action of components from widow spiders’ venom and eggs. The recombinant latroeggtoxin-V inhibits the proliferation and migration of breast cancer cells (MDA-MB-231) and also causes their apoptosis; the toxin inhibits the activity of the Na^+^/K^+^-ATPase in a concentration-dependent manner, suggesting that its action affects ion transport [218]. In a short communication, Mousavi *et al*. [219] reported that *L. dahli* crude venom induces cell death in a breast cancer cell line (MCF-7), possibly by apoptosis. Rivera-de-Torre *et al*. [220] have proposed that conjugating spider toxins with antibodies that target tumor cells could enhance the selectivity of the toxins toward the tumor. Pore-forming toxins such as α-LTX are ideal candidates for this strategy, as they deal damage to the cell without the need for internalization. Although a deeper understanding of the mechanisms of toxins and putative immunotoxin complexes is still required, this strategy could represent an advance in cancer treatment.

### Novel ICK toxins from widow spider venom

As discussed in the section “proteome and transcriptome analyses of *Latrodectus* venom”, all of the studies performed have reported the presence of ICK-motif toxins, which in spider venoms are cystine-stabilized peptides with a specific pattern of four sequential disulfide bridges (C1-C4, C2-C5, C3-C8, C6-C7). The presence of these toxins was unprecedented in the Theridiidae family until He *et al*. [207] reported that *L. tredecimguttatus* venom contains ICK peptides with sequence homology to members of the CSTX superfamily; similar results were later found for *L. hesperus* [209] and *L. mactans* [211]. CSTX-1 has been reported to selectively block L-type Ca^2+^ channels in neurons [221] and to exert cytolytic activity on both prokaryotic and eukaryotic cells [222]. Molecules that are structurally related to CSTX-1, such as the novel ICK peptides from *Latrodectus* spp., are likely to promote similar biological activities. ICK toxins can act as hemolytic, antiviral, and antibacterial agents as well as ion channel blockers [223, 224]. Moreover, the ICK motif gives stability to the molecule, a very important characteristic for molecular engineering applications [224]. Hence, ICK toxins from widow spiders can potentially be used as pharmacological tools for multiple studies.

## Conclusion

Although the real epidemiological profile of latrodectism in the Americas is still unknown, the reports of envenomation by *Latrodectus* spp. date back to the 18^th^ century. Widow spiders from the Americas encompass 44% of the valid *Latrodectus* species, yet the venom from most of these species remains uncharacterized. Most of the data obtained from venom analyses focus on latrotoxins and latrodectins from a few species. For example, α-LTX is the most studied toxin so far. It is known to play an important role in envenomation by inducing massive non-specific neurotransmitter release. Recently, high-throughput methods have been used in the study of widow spider venoms, allowing a deeper comprehension of the molecules present in *Latrodectus* spp. Nevertheless, the current knowledge on the subject is still limited, so the exploration and comparison among different venoms are necessary.

Future studies should focus not only on the venom, but also on the silk fibers, eggs, and body parts. This way, not only a better understanding of the envenomation picture would be possible, but also new molecules of biotechnological interest could be uncovered. Moreover, these analyses could clarify the phylogenetic relationships within the *Latrodectus* genus and among widow spiders and other Araneae. Lastly, even though the use of antivenom in the treatment of *Latrodectus* bites is still controversial, we believe that the production of a Pan American anti-*Latrodectus* serum would be beneficial from a public health perspective, given the good results of serotherapy reported in the literature. However, more studies are necessary to reduce the immunogenicity of antivenom and therefore the chances of unwanted side effects.

### Abbreviations

Ach: acetylcholine; ACVGM: aqueous components of venom glands macerated; CHH: crustacean hyperglycaemic hormone; CIRL: calcium-independent receptor of α-LTX; CRISPs: cysteine-rich secretory proteins; CSTX: *Cupiennius salei* toxin; GABA: γ-aminobutyric acid; GIH: gonad-inhibiting hormone; ICK: inhibitor cystine knot; LCT: latrocrustatoxin; LIT: latroinsectotoxin; LPH1: latrophilin 1; LRR: leucine-rich repeats; LTXs: latrotoxins; MIH: molt-inhibiting hormone; nAChR: nicotinic acetylcholine receptor; NMJ: neuromuscular junction; NXR-Iα: neurexin-Iα; PKC: protein kinase C; PLC: phospholipase C; PTPσ: protein tyrosine phosphatase σ; SV: synaptic vessels; VGCC: voltage-gated calcium channels; VIH: vitellogenesis-inhibiting hormone; α-LIT: α-latroinsectotoxin; α-LTX: α-latrotoxin.

## Footnotes

1 To allow the comparison between works, the LD50 values reported by De Roodt et al. [101] were recalculated and expressed as mg/kg, considering 20 g as the mean weight of mice.
